# Why are they “unreached”? Macro and Meso determinants of health care access in hard to reach areas of Odisha, India

**DOI:** 10.1186/s12939-022-01817-y

**Published:** 2023-01-05

**Authors:** Srinivas Nallala, Upasona Ghosh, Shyama Sundari Desaraju, Shridhar Kadam, Rahul Reddy Kadarpeta, Sara Van Belle

**Affiliations:** 1 Indian Institute of Public Health Bhubaneswar, Bhubaneswar, India; 2 Health System Transformation Platform, New Delhi, India; 3 Institute of Tropical Medicine, Antwerp, Belgium

**Keywords:** Hard to reach, Tribal health, Geographical intersectionality, Social determinants of health, Health inequity, Vulnerable population

## Abstract

**Background:**

Reaching hard to reach populations is key to reduce health inequities. Despite targeted interventions, status of crucial public health indicators like neonatal and maternal mortality is still far from optimal. Complex interplay of social determinants can influence both communities and health care workers to effectively access each other. We argue that culturally sensitive and contextually relevant healthcare provision has potential to increase health care utilization by the vulnerable communities living in remote areas.

**Methods:**

The study is an exploratory case study using rapid ethnographic techniques to understand the interplay of social determinants in hard to reach areas of Odisha state, India. We used in-depth interviews, focus group discussion, participatory action research and key informant interviews as tools for data collection. The analysis of data has been guided by thematic analysis approach.

**Results:**

We found that there are further layers within the designated hard to reach areas and those can be designated as-i) extremely remote ii) remote and iii) reachable areas. Degree of geographic difficulties and cultural dynamics are deciding the ‘perceived’ isolation and interaction with health care providers in hard to reach areas. This ultimately leads to impacting the utilization of the facilities. At extremely remote areas, felt health needs are mainly fulfilled by traditional healers and ethno-medical practices. In reachable areas, people are more prone to seek care from the public health facilities because of easy accessibility and outreach. Being in middle people in remote areas, diversify health care seeking depending upon social (e.g. patient’s gender) economic (e.g. avoid catastrophic expenditure) and health system (timely availability of health human resources, language barriers) factors.

**Conclusion:**

Our research highlights the need to value and appreciate different worldviews, beliefs and practices, and their understanding of and engagement with the pluralistic health care system around them. Other than pursuing the ‘mainstreaming’ of a standardized health system model across hard to reach areas, strategies need to be adaptive as per local factors. To handle that existing policies need revision with a focus on culturally sensitive and contextual care provision.

## Introduction

Reaching hard to reach populations is key to reduce inequities, one of the objectives of the SDGs, and is essential for policymakers around the world, to ensure socially just, people-centered care [1, 2]. This implies providing access to responsive, inclusive and people-centred health services regardless of where people live [3]. However, even when the infrastructure, human resources and logistics are in place, health systems are sometimes not able to offer accessible and acceptable care to all due to the complex interplay of social determinants. These social determinants vary contextually and originate at intersecting levels. Determinants can be at individual level (micro), it can be at the level of health care provision (meso) or at the wider societal level (macro) within a given context [4, 5].

Intersectionality, with its roots in Afro-American studies [6], has made inroads in health policy and systems research as a theoretical lens to explore interactions between social determinants such as ethnicity, gender and religion and how the confluence of these determines health outcomes [7]. Following an intersectionality lens, the interplay between determinants like geographic location or “place” [8–10], ethnicity [11], and poverty [12] determines the “capabilities” of a population [13], and their ability to receive health services, which in turn influences their health and well-being outcomes. Moreover, determinants rooted in social structure and culture, such as historical residential segregation, continues to influence people’s access to health services.

Strengthening the collective capabilities or agency of disadvantaged communities within a given social context is a long-term process as it requires changing power structures, or the redistribution of resources, between ethnic minorities and a given majority population. In such a context, the provision of health care can be a lever to empower communities to access health care [14].

Our paper presents the case study of socio-geographically hard to reach communities from the Rayagada district of Odisha state, India. In this paper, we present qualitative evidence on how social, cultural and geographical factors are determining the health seeking behaviour of ethnic minorities living in that particular remote region. We argue that the provision of “embedded health care”, defined here as culturally sensitive and contextually relevant care. Embedded health care has the potential to increase the health care utilization of communities living in remote, mountainous areas [6, 15, 16].

Odisha, a low-income state in Eastern India, has undergone significant reforms to close gaps in health inequalities by focusing on health governance, including organizational restructuring, and health financing reform. Among the approximately 42 million [17] population of Odisha, the Scheduled Tribes and Scheduled Castes comprise 23 percent and 17 percent respectively, a traditionally marginalized section of Indian society. According to Subramanian, [18], “indigenous status in the context of India was operationalized by Indian government by way of the category of scheduled tribes, or “Adivasis”, which refers to people living in tribal communities characterized by distinctive social, cultural, historical, and geographical circumstances”. Approximately half of Odisha’s population is under the poverty line,^1^ with limited access to resources due to a complex interplay of social, economic, cultural and geographical dynamics. Majority of those under the poverty line are Schedule Tribes and Scheduled caste [17]. Furthermore, frequent cyclones, floodings and dry spells not only impoverish families but also push the already marginalized communities further into poverty [19–21].

Odisha has geographical barriers like the Niamagiri hill range, 61,204.17 sq km of forested areas which make 13.5% of the state’s total health facilities hard to reach [22]. Hard to reach areas are mainly inhabited by some of the Particularly Vulnerable Tribal Groups (PVTGs)^2^ like the *Dongria Khond, the Saharia, and the Lanjia Soura,* who are the most impoverished among the tribals. Their main livelihood is step farming and collection of natural resources from the forest. Despite the state government’s continuous efforts, the state’s health indicators are indicative of ongoing challenges primarily in relation to the health system, as the neonatal mortality rate [40] and maternal (168) mortality ratios indicate [23]. Furthermore, the health system of Odisha in tribal inhabitated areas is challenged by lack of health infrastructure including trained health human resources at primary, secondary and tertiary care level [24, 25].

In order to ensure service provision for the PVTG living in hard to reach and socio-economically disadvantaged areas, Odisha’s state’s Health and Family Welfare department implemented specific health system strengthening initiatives regarding the management of human resources and primary care provision, by involving civil society organizations through contracting out of service provision in some of the harder to reach pockets. One of the government initiatives to bolster geographical equity in health care provision in Odisha was the implementation of categorization of health facilities into five vulnerable categories (V0, V1, V2, V3 and V4). Following criteria were taken into account while categorizing the health facilities: [1] the health facility being located on difficult terrain, [2] population of tribal groups in the area, [3] political unrest, [4] availability of road and transport infrastructure, [5] availability of social infrastructure (e.g. number of schools), and [6] distance from the state headquarters. About 13.5% of health facilities in Odisha belong to the V3 and V4 regions for which the state came up with targeted interventions like compulsory posting of medical doctors for three years at the beginning of their career, a time-bound transfer and place-based incentives to increase the retention of medical doctors. They also created NGO-managed PHCs through a contracting- out mechanism, bike ambulances and maternity waiting homes. Furthermore, they converted some of the PHCs to Health and Wellness Centres (HWCs) in addition to upgrading all of the subcentres to HWC under the 2017 national health policy.

Despite these targeted interventions, outcomes have not yet been achieved [26]. This study, therefore, aimed to reconnaissance/investigate why these hard to reach areas remain “unreached” despite policy initiatives to strengthen the health system and primary health care. The study explored how the dominant narrative of quantitative evidence [26, 27] often leads to top-down technical solutions without sufficiently taking into account the contextual complexity. The study also tried to come up with plausible recommendations for embedded healthcare provision. The paper thus presents empirical evidence on the complex interplay of determinants contributing to the health outcomes of ethnic minorities in hard to reach areas and a better understanding on how to tackle health inequities in areas with socio-geographic intersectionalities as main / key health system barriers.

## Methodology

We opted for an exploratory embedded case study design [28]. Exploratory case studies provide insights to public health challenges which can inform future programme design [29].

We conducted a rapid ethnography [30] as part of a larger project on diagnostics for health system strengthening in Odisha State, funded by Tata Trusts. We used in-depth interviews, focus group discussions, participatory action research, and key informant interviews as tools for data collection. This blended approach ensured data triangulation. Interview schedules and topic guides were developed for conducting interviews with key informants in the community, like community leaders, traditional healers, frontline health workers, and community-based organizations working in tribal health domain. Data collection was conducted in two phases with an analysis phase in between. Initial findings were analyzed to explore emerging themes with the objective of identifying gaps in community access to health care. A total of 108 in-depth interviews and 14 focus group discussions were conducted to cover hard to reach areas in the selected districts. Ethical Clearance was obtained.

Most of the authors of the project have been advising state governments as technical experts for close to a decade. Being involved in many government-led public health interventions made them understand the resource constraints faced by state governments in terms of both financial and human resources for health. While conducting this study to diagnose access barriers for further strengthening the health system as independent researchers, they used reflexive self-observation techniques to overcome their dilemmas as former policy advocates of the state government [31]. Periodic inter-team discussions were helpful to reflect on the community’s perspectives and how this differs from the state government’s view.

Though everyone in the field data collection team had significant experience working in remote rural settings in various Indian states, difficulties in actually reaching some of the more remote villages in this setting put the members in shock at first and presented them with a moral dilemma about what could be feasibly suggested for improving the health situation. The immediate solution would be top-down infrastructural development like roads, health facilities, transport, and communication networks. However, as the research progressed, the team gained a better understanding of socio-cultural issues and began exploring with the communities and key informants on how health care could be more culturally sensitive and adapted to this context in particular [32].

### Selection of study district and villages

The Rayagada district, which has a population of nearly a million people, is one of the most difficult to reach districts in Odisha, with approximately 74.5% (V3 + V4) of the health institutions classified as “hard-to-reach” by the state authorities. Ethnic groups, especially those considered “Particularly Vulnerable Tribal Groups” (PVTGs) by the state authorities^3^ made Rayagada a suitable case for our study objectives.

A key stakeholder consultation was conducted with the district health officials of Rayagada to select the villages. They classified villages as hard to reach based on: 1) the distance from the highway leading to district headquarters, 2) the presence of communication, 3) the presence of a local high school, 4) the presence of a Panchayat member^4^ and 5) finally, the number of seasonal or permanent transportation cut-offs. Respectively three blocks, with an average of 100,000 population per block, with the highest number of hard to reach villages, and three blocks with the lowest number of hard to reach villages were selected to explore differences in nested socio-geographic vulnerabilities in terms of access to health care.

### Collection of data

In each hard-to-reach village, the data collection process started with a transect walk [33] aimed at understanding geographic and socio-economic characteristics, as well as the available health care resources. During the transect walk, informal discussions with community members helped to build the necessary rapport with the community and identify prospective informants for the focus groups. The learning and understanding gained from the transect walk were later converted into a health resource map identifying the distances between multiple healthcare providers and facilities in consultation with community members. The map helped to understand the existing health care delivery context, both formal and informal providers, of that particular village. Community-level key informants and suitable participants for in-depth interviews were mutually identified by the researchers and community members during the transect walk. The focus group discussions were conducted with a mixed group of participants (male and female with an age range of 25 to 50) following a guide.

In-depth interviews were conducted with women of reproductive age from the same villages on the issues such as socio-cultural practices in the community especially around menarche, pregnancy, childbirth, care seeking behaviour, ethno-medicine, perceptions about the health system etc. In key informant interviews with health providers (Table 1), we explored their views on communities, health system responsiveness, and the perceptions of the providers regarding the geographical, social and system-related barriers were discussed.

**Table 1:** Study participants and sample size

Sl. No	Facility level	Study participants	No. of participants	Total No. of interviews	Total No. of FGDs
1	District	CDMO, DPM, RCH Consultant, Immunization consultant, ASHA manager	5	5	0
2	Block	BPM, MOIC	7*2	14	0
3	PHC	Medical officer	7*2*1	14	0
4	SC	ANM/MPHW (M)/ASHA	7*2*1	14	0
5	Community	Community members	7*2*1*4	56	7*2
6		Traditional healer	5	5	0
	**TOTAL**			108	14

### Data analysis

All interviews, focus group discussions, and field diaries of researchers were translated to English. The data were then uploaded into ATLAS.ti software version 8 for analysis and data management. The analysis of data has been guided by a thematic analysis approach [34, 35]. A coding matrix was developed, and transcripts were then coded under three major themes: i) cultural practices, ii) geographic barriers, and ii) health care provision, which were further divided into various sub-themes. From the transect walk, we calculated i) the distance to the residence of ASHA; ii) the distance to nearest health facility, iii) the distance to a motorable point from the community.

## Results

After a decade of implementing policy and programs targeted to ‘hard-to reach’ areas, geographical inaccessibility has improved which in turn resulted in improved health access. However, we find a spectrum of geographic as well as socio-cultural barriers are still there, intersecting with each other and influencing people’s access to healthcare.

### Differential access and care-seeking within the ‘hard to reach’ areas

The study district Rayagada has the highest number of ‘hard to reach’ health facilities^5^ compared to other districts in Odisha state. However, the study found that the access to health facilities is highly variable across the district. On the basis of geographic remoteness and communication access, the study found three types of ‘hard to reach areas’—1) reachable, 2) remote and 3) extremely remote areas.

The reachable areas are those that have concrete roads, connectivity and primary and/or secondary schools, and access to a primary health care facility. Whereas, remote and extreme remote areas are located in hilly and forest terrain and mostly inaccessible. Remote areas, located in hilly and wooded terrain, are within the proximity of about two to five km from the nearest motorable road. A few remote areas could be accessed by a two-wheeler. Some of these regions have seasonal cut-offs, especially during monsoon. Primarily, Schedule Tribes and Scheduled Caste communities reside in these locations. Basic infrastructure such as telecommunications /internet and electricity are scant in these parts. Extremely remote areas are located at a distance of more than five kilometers and up to 20 km from the nearest motorable road and can only be accessed by foot due to the hilly and forested terrain. The villages here are scattered and mostly located on top of the Niyamagiri hill^6^ range. These areas are primarily inhabited by Particularly Vulnerable Tribal Groups (PVTGs) and lack basic infrastructure. There is limited outreach by frontline health providers.

**Fig. 1:**
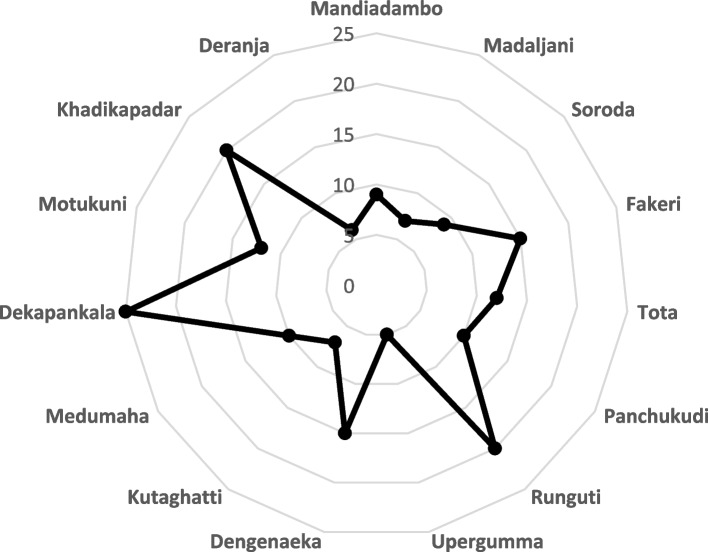
Distance of Villages from nearest health facilities

In the course of our transect walk, we collected data on the distance to the nearest public health facility from the study villages. We observed that the range of distance varies from 5 to 20 km (Fig. 1) depending on their respective location in reachable, remote and extremely remote areas.

Obviously, people in villages from extremely remote areas have less options for seeking formal health care. Communities in these villages tend to seek care from traditional healers as a first point of care and are also sub optimally reached by Accredited Social Health Activist and Auxiliary Nurse Midwife.^7^ These findings are congruent with studies in other remote parts of various states of India such as Jammu and Kashmir, Tamilnadu and Andhra Pradesh [36–39].

According to a female respondent of one of the villages under study:

“The road condition is terrible and carrying a seriously ill person is very difficult. If anyone suddenly falls ill or is critical, it is challenging to take them to the hospital immediately. To go somewhere, we need to walk long distances on harsh road"—female respondent from village 10.

Villages which are situated at reachable points, have much better access to public health facilities. A fair connection with motorable points, easy access to public health transportation and regular outreach services by ASHA and ANM make public health care seeking easier in these parts [37, 40].

Remote areas have multiple providers, both formal and informal, influencing the care-seeking behaviour of the communities. A wide range of care-seeking behaviour was observed, from visiting traditional healers and faith healers to accessing public first line health facilities. Our findings confirm the previous findings that communities often switch providers depending upon availability, seasonal geographic access, perceived severity of the illness and opportunity cost [36, 41, 42]. The remote and extremely remote areas have a higher concentration of informal providers, traditional and faith healers with significant utilization of their services compared to reachable areas. Study findings indicate that even though people are aware of sub-optimal quality of care provided by the informal providers, they still have to seek care from them because overcoming geographical barriers incurs additional costs. Respondents expressed that as private road transportation is quite infrequent on these routes, people have to hire transportation which often increases out of pocket expenditure. People also mentioned the long waiting times at the facilities, further adding up to their opportunity cost of seeking care from a public health facility. According to a male respondent from a village:

"We face a lot of difficulties to reach a health facility. We have to walk long distance in that weak condition to reach the motorable point and then again wait long hours for vehicle. It's not like that I would reach, and a vehicle would be there for me to buy ticket of 10- 20 INR and go".

According to a female respondent during an FGD in one of the remote villages:

“To call the vehicle, we have to go down. It will take 1 to 2hrs for the vehicle to reach. In all this, we waste our crucial time. We feel, why to face so much of challenges to go to hospital, so prefer to deliver at home".

**Fig. 2:**
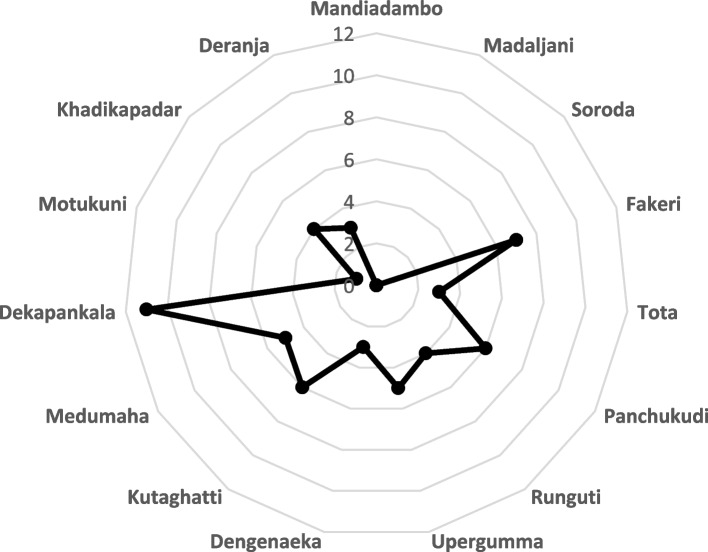
Distance of ASHA residence from study villages

The geographical location of a village is significantly determining the health seeking behaviour of its people, impacting on the utilization of formal health facilities. Moreover, the residence of the ASHA in most of the studied villages is at a distance, which might be a challenge to access antenatal care services. Figure 2 shows that the ASHA reside more than five km in nine villages out of the 15 villages. Hence, close monitoring of high-risk pregnancies is not always possible. The number of beneficiaries attending an ANC session is also usually low following the frontline health worker respondents.

### Disconnect in access to services despite infrastructural development

**Fig. 3:**
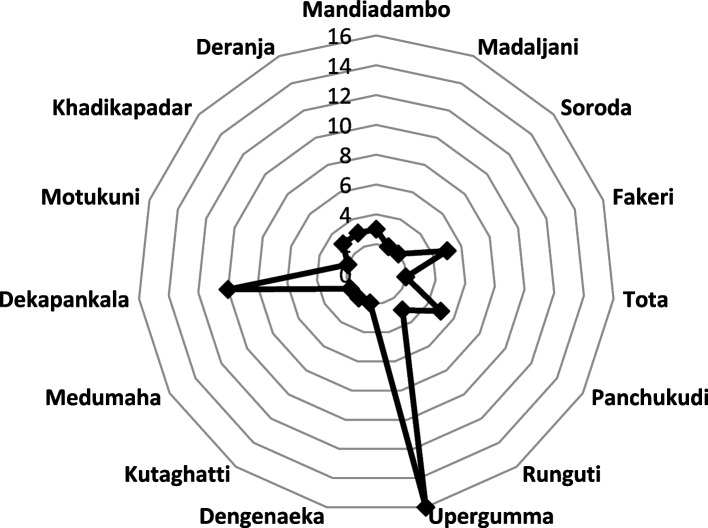
Distance of the selected villages from motorable point (in KMs)

The study district has seen infrastructural development in recent years and became connected through various roadways and two bridges with the neighbouring districts under various State and central government schemes. However, the road coverage and transportation system are still limited in all of the study areas and there is still a disconnect with primary health care health facilities. To partially address this, the state has come up with the concept of "motorable point", where an ambulance or other transportation service can be made available. However, following the communities, many of such motorable points do not have public transportation facilities connecting them to the villages. In effect, distance to the nearest motorable point varied widely across the villages, especially in case of the extremely remote ones (Fig. 3).

Walking for more than 10 km to access health services is common among the people residing in the extremely remote areas. Apart from the challenging terrain until the motorable point, the limited transportation options from the foothills increases the waiting time for people at facility as they arrive late. The whole process of seeking care consumes an entire day that results in loss of wage, increased expenditure on food and transportation, in addition to the physical stress.

"We face a lot of difficulties to reach to the motorable point. Either we have to walk long distance in that weak condition or hire a private vehicle, if available to reach the motorable point and then again wait long hours for the public transport. It's not like that I would reach, and a vehicle would be there for me to buy ticket of 10- 20 INR and go"—A female respondent from village 2.

Further, during emergencies, community members had to borrow money from friends to hire a private vehicle for transportation to the health facility. According to the respondents, when it is not emergency, they prefer not to visit public health facilities due to the high opportunity cost. The majority of the community members and health staff indicated that people visit the public health facility mostly on weekly market day, as it is both convenient as well as cost-effective.

Along with lack of transportation, emergency services, like ambulance services, are the most affected due to poor t connectivity in the regions. From extremely remote and remote areas, information could not be provided in time to the ambulance, hampering emergency. According to the respondents, even if both ambulance and patient are in the same village but in different locations, it can be challenging to communicate their exact location.

“To call the ambulance, we have to go down from the hill. It will take 1 to 2 h for the ambulance to reach here. During this process, we waste our crucial time. We feel why to face such problems to go to the hospital? It is better to deliver at home”-stated by a female respondent during a FGD in village 5. Hence, despite government’s constant effort to improve the road infrastructure, timely access to health facilities remains a challenge for people living in the remote and extremely remote areas.

### Seeking care: transition between ethno-medicinal practice and formal care

Despite policy measures, indigenous communities in India still face health inequalities than their non-indigenous counterparts. As a dominant narrative by the health implementers, traditional beliefs and practices of indigenous communities are considered as the main reason for their sub-optimal access to formal health care. However, recent studies mention the lack of culturally adapted health services and the lack of negotiation power of indigenous people as barriers to access public health care [18, 43]. Our study findings confirm that targeted interventions for hard to reach areas lack consideration do not take the socio-cultural sensitivity of services into account. Moreover, health care providers from outside the region, are not being trained in culturally sensitive service delivery, which further widens the gap in access.

The majority of the study participants belong to Particularly Vulnerable Tribal Groups such as the *Dongria Khond* and the *Saba*r communities who have their specific set of beliefs, customs, rituals and practices related to health and illness. For those indigenous communities living in extremely remote areas, subsistence is a critical aspect of life and any physical condition which is hampering that subsistence process is considered as a disease. Severe clinical manifestations or symptoms like high fever, dizziness, vomiting is considered to be prominent symptoms of illness. Communities, mostly from the extremely remote areas, believe supernatural powers to be the cause of the diseases; thus, they mainly resort to traditional treatments. Faith-healing and home remedies from plants, roots and tubers from the forests and hills, form a significant part of the health care system of these indigenous communities (Table 2). *“If we don’t have a fever, we believe we are fine. We suffer from fever due to ghosts, which we get from Dangar (i.e. forests) while working there”*- stated by a female respondent from village 6.

**Table 2:** Causes of sub-optimal access (providers and communities)

Community	Frontline health workers
Belief in natural resources	Lack of awareness
Traditional MCH practices	Traditional practices
Ethno-linguistic barriers	Ethno-linguistic barriers
Lack of trust on public health system	Non-acceptance of the communities
Opportunity cost to access care	Transport and safety issues
Geographic hurdles	Geographic hurdles

This embedded view was contrary to the perceptions of the health care providers who saw these beliefs as one of the principal barriers in convincing indigenous communities to utilize formal health care. *“Some people, even if they are suffering severely, they think it is to be an effect of some evil eye and go to a faith healer. They trust the faith healer more than ASHA. If she asks them to go to the hospital and get the tests done, they say, they are affected by the evil eye; hence no need of a doctor”*- stated by a medical officer of a studied block.

Communities of hard to reach areas consider pregnancy and child birth a natural event which does not require medical intervention [26]. Pregnant women reportedly participate in strenuous activities like fetching water, wood collection, household and cultivation work throughout their pregnancy. The female respondents were unanimous that the burden of livelihood mostly falls on women. Hence, women prioritized their work rather than their health, even during pregnancy.

“We follow our traditional practices during the pregnancy. During that period, we do all types of works like bringing water, working in ‘Dangar’ (Agricultural works), cutting trees etc. along with the household chores.”—stated by a female respondent from village 8.

According to the health providers, home deliveries are not only typical of remote and extremely remote areas, but are also common in villages of the reachable areas. Frontline health workers indicated that due to targeted interventions like the creation of maternity waiting homes and the repeated sensitization of health staff, the public health system has been able to make some inroads within the reachable areas. However, there remain considerable challenges to mobilize people from remote and extremely remote areas for institutional deliveries due to their socio-cultural beliefs. As the process of delivery is considered to be ‘impure’ by the community, a pregnant woman should not be touched by anyone during and after the process of delivery, except her mother. *“A pregnant woman would be kept in isolation near to her labour. There would be nobody to take care of her, because of which she usually doesn’t get the required medical help, not even from the male quacks available nearby”*- stated by the ASHA worker from the same village.

Post-delivery, cultural practices such as the cutting of the umbilical cord with sharp head-gear; the bathing of mother and baby immediately after delivery; isolated staying mother and the new-born in the backyard of the house, the application of various home remedies were considered by health providers as root causes of neonatal and maternal morbidity.

### Gender as a significant influencer of care-seeking behaviour

Since the responsibility of household and livelihood rests on women, she often ends up neglecting her health and well-being. Innovative interventions like maternity waiting homes face implementation challenges mainly due to a disregard for gender roles: when a woman is responsible for household chores, taking care of children and earning a livelihood, she is not allowed to waste time in waiting homes [26].

Where delayed care-seeking is a critical issue for women, men’s negligence of health is also an important concern. The health staff in most of the studied villages indicated that very few men actually seek care. Since the men, especially from the tribal community are in an inebriated state, they often ignore their ailments. Another possible explanation might be that various frontline providers like ASHA and ANM, were more capable of mobilizing women because they themselves are women. Men in general might be ignored by the “maternal and child care” lens of the health system and gender norms of the society. “ASHAs can mobilize females properly but they are often unable to do the same in case of males due to strict gender norms of the society”- stated by a health official from one of the studied blocks.

Also, in case of women’s care seeking, the study found that women internalized their secondary status by opting not to seek care after the spouse’s objection. Critical health care seeking decisions were taken by the man of the house, who is mostly out of the radar of the female-oriented frontline health care delivery system.

### Ethno-caste dynamics: barrier in accessing targeted facilities

The degree of geographic difficulties and cultural dynamics of the villages bear upon interaction with the health care providers in hard to reach areas Many of the health worker respondents identified the caste system as a crucial barrier in providing health services to the people residing in hard to reach areas. There is a clear-cut divide between social categories living in the same region where both Scheduled Caste and Scheduled Tribe people consider other group to be untouchables. Moreover, “Other Backward Class” people consider both SC and ST to be untouchables. Issues arising out of these ethno-caste dynamics are mostly related to using water and sanitation facilities and food preparation at government ICDS centres.^8^ In the few areas where the ST population dominates, SC people are not allowed to specific water sources and food outlets. *“It is a traditional practice. We do not mix. Their ‘dangar’ and our ‘dangar’ are also in different areas”*- stated by a female respondent from one of the studied blocks.

The brunt of this is being carried by the health staff working in those areas. In some locations, communities refuse to take services from providers which do not belong to the same caste. In reachable places where “Other Backward Classes” reside, they are higher up in social ladder. Hence, they are reluctant to use services which they have to share with the SC and ST people.

The medical officer from one of the study block shared his experience regarding these crucial social determinants—*“We had seen a severely anaemic mother who needed blood transfusion. However, she was totally rigid to go to the hospital. That patient belongs to ST category whereas the ASHA was a SC person. Hence, she refused to go with her”.*

## Discussion

This study examined the interactions between geographical and socio-cultural determinants of care seeking behaviour of communities living in hard to reach areas of Odisha state—a vulnerable region with its own socio-geographic complexities. Salient determinants demonstrate similarities with other hard to reach parts of India but differ in intensity and outcome. Our findings appear to indicate that a geography-focus blanket approach to equitable care provision is missing the dynamics of community care seeking and inter-linkages between the two which are impacting the health and wellbeing of the communities living there [26].

We found that socio-geographic factors are creating a divide within the hard to reach areas between formal care and socio-cultural practices. To tackle this divide, existing policy initiatives need revision with a focus on more culturally sensitive care provision.

The Report of the Expert committee on Tribal Health of government of India published in 2018 [44] recommended to revisit health interventions in tribal regions to make them more culturally sensitive. Our study generated findings in this regard on how to operationalize policy recommendations which can potentially support Odisha government’s targeted policies on health of hard to reach areas. The findings urge the requirement of such cultural sensitization of health care provisioning along with the revisiting the definitive boundaries of hard to reach.

Moreover, our findings echoes policy shift towards people-centred health system in Odisha state and the regions alike which was also stated by Contractor et al. in 2018. Over all, the study generated evidence beyond the scope of hard to reach areas and further demonstrated the transitionary phase of India’s tribal health from ethno-medicine to formal health care for which they need conscious effort from the health system to become more socio-culturally inclusive. Findings confirm Guite, N., & Acharya, S and George and Iyer 2006 of a more pronounced medical pluralism in remote areas [45] as people in remote areas knowingly opt for traditional healers [46].

Culture and social systems are dynamic and subject to constant flow [47]. This study demonstrates how the ST and PVTGs have been adapting their health seeking behaviour while living in a hard to reach area. A major catalyst in this process is their exposure to or their interaction with the public health system through the CHW. The study points out that traditional health care has its own strategy of survival and adaptation in a rapidly changing environment. Traditional medicine and healing practices provide a much-needed linkage between communities and their natural living environment. A similar connection with the public health system can be difficult to establish for tribal groups if the culturally embedded protection of services provided by traditional healers is not taken into account. Traditional healers being an integral part of the community, could potentially play a role as to increase acceptance and trust of the public health system, resulting in greater services uptake. Structural hierarchies are at the root of the mistrust of the public health system leading to a dissonance between the felt needs of communities living in hard to reach tribal areas and the services offered by the health system [26, 48, 49]. This paper proffers the argument against generalizing health needs of indigenous communities to be integrated into a mainstream rural health system through large quantitative generalization. We build on previous work that quantitative generalization influences policy making [27] which often leads to ‘oversimplification’ and a mere ‘technical’ solution in the domain of maternal and child health [26].

## Conclusion

Despite socio-geographical challenges and contingencies of communities living in hard to reach areas, the topography and socio-cultural practices of indigenous communities in Odisha are not insurmountable barriers to effective care seeking. On the contrary, they can facilitate the creation of ‘locally owned’ solutions at the intersection of socio-geographic conditions, social relations, and cultural practices that frame the health system ecology which is at the heart of health of Odisha’s hard to reach areas.

Hence, the definition of hard to reach areas used by the state, needs to be revisited with a more nuanced understanding of the context. The context, including socio-geographical hierarchies and local and state-local power dynamics intersects the health system [50]. Hence, it requires specific culturally sensitive planning and programs which can broaden up the reach of the health system. Our results suggest that the Odisha state’s geographic vulnerability criteria need to be revised. Also, one needs to reconsider the “vulnerability” discourse. Indigenous communities residing in hard to reach areas will always considered to be ‘vulnerable’ from a ‘bureaucratic logic’ [51] which indicates a top-down policy making process without the involvement of the local perspective.

Our research also highlights the need to valorize and appreciate the richness inherent in different worldviews, beliefs and practices, and their understanding of and engagement with the pluralistic health care system around them. Rather than pointing at them for delaying formal care as in the 3 delays model, [52] there is a need to consider actual health care seeking pathways. Working with family members, like the mother in law, as well as other community gatekeepers like traditional healers, must be done in a culturally sensitive way. For example, facilitating prompt care seeking should be done in conjunction with empowering a young generation of mothers, so as not to further reinforce existing power hierarchies. Finally, an improved referral network with the engagement of key actors in the transportation sector is an urgent priority for hard to reach areas. Just as access to health care is an urgent need in this region, so is the quality of care and the accountability towards communities. Other than pursuing the ‘mainstreaming’ of a standardized health system model across hard to reach areas, strategies need to be culturally adaptive and contextually specific. We can only transform the health system through mediating existing actors and resources.

## Data Availability

All the data collected and analyzed in this study are available with the corresponding author and would be provided upon request.

## Footnotes

1 BPL: According to Tendulkar Commiitte report, 2004–05, the families which spend 32 INR /person/ day in rural areas and 47 INR/person/day in urban areas come under BPL (Below Poverty Line) category.

2 PVTGs are less developed among the tribal groups in Indian context. The Dhebar commission created PVTGs as a sub-category among the tribals in 1973 on the basis of geographic isolation, slower development and traditional ways of livelihood.

3 PVTGs constitutes the most vunerable sections among the tribals and inhabit isolated, remote, and difficult areas in small and scattered hamlets/habitats (Ministry of Tribal Affairs,Government of India).

4 Panchayat is the system of local self-government of villages in rural India.

5 We have selected a geographically remote district with the criteria of number of health facilities in hard to reach areas as per the state government’s parameters.

6 The Niyamgiri is a hill range situated in the districts of Kalahandi and Rayagada in the south-west of Odisha, India. These hills are home to Dongria Kondh indigenous people.

7 Accredited Social Health Activist (ASHA) – is a trained female community health activist under National Health Mission, provided to every village. Selected from the village itself and accountable to it, works as an interface between the community and the public health system; Auxiliary Nurse-Midwife (ANM) - is a health worker based at a sub-center and visits villages in addition to providing care at the subcentre.

8 Integrated Child Development Services is a government program in India which provides nutritional meals, preschool education, primary healthcare, immunization, health check-up and referral services to children under 6 years of age and their mothers.
